# Integration of individual and social information for decision-making in groups of different sizes

**DOI:** 10.1371/journal.pbio.2001958

**Published:** 2017-06-28

**Authors:** Seongmin A. Park, Sidney Goïame, David A. O'Connor, Jean-Claude Dreher

**Affiliations:** 1 Neuroeconomics, Reward and Decision-making Team, Institut des Sciences Cognitives Marc Jeannerod, Centre National de la Recherche Scientifique, Bron, France; 2 Université Claude-Bernard Lyon 1, Villeurbanne, France; University of Oxford, United Kingdom of Great Britain and Northern Ireland

## Abstract

When making judgments in a group, individuals often revise their initial beliefs about the best judgment to make given what others believe. Despite the ubiquity of this phenomenon, we know little about how the brain updates beliefs when integrating personal judgments (individual information) with those of others (social information). Here, we investigated the neurocomputational mechanisms of how we adapt our judgments to those made by groups of different sizes, in the context of jury decisions for a criminal. By testing different theoretical models, we showed that a social Bayesian inference model captured changes in judgments better than 2 other models. Our results showed that participants updated their beliefs by appropriately weighting individual and social sources of information according to their respective credibility. When investigating 2 fundamental computations of Bayesian inference, belief updates and credibility estimates of social information, we found that the dorsal anterior cingulate cortex (dACC) computed the level of belief updates, while the bilateral frontopolar cortex (FPC) was more engaged in individuals who assigned a greater credibility to the judgments of a larger group. Moreover, increased functional connectivity between these 2 brain regions reflected a greater influence of group size on the relative credibility of social information. These results provide a mechanistic understanding of the computational roles of the FPC-dACC network in steering judgment adaptation to a group’s opinion. Taken together, these findings provide a computational account of how the human brain integrates individual and social information for decision-making in groups.

## Author summary

In collective decisions, both the size of groups and the confidence that each member has in their own judgment determine how much a given individual will adapt to the judgment of the group. Here, we show that judgment adaptation during collective decisions—a fundamental brain mechanism needed for fluid functioning of social organizations—can be accounted for by Bayesian inference computations. At the time of judgment adaptation, individuals trade off the credibility inferred from their own confidence levels against the credibility of social information. The dorsal anterior cingulate cortex (dACC) represented belief updates, while the lateral frontopolar cortex (FPC) monitored the changes in credibility assigned to social information. These results provide a neurocomputational understanding of how individuals benefit both from the wisdom of larger groups and from their own confidence.

## Introduction

When making decisions in a group, individuals can adapt their initial beliefs according to the social influence produced by the opinions of other individuals in the group. In modern society, this type of process is widespread and can be seen in settings ranging from work meetings to the courtroom. In these instances, individuals are asked to come together to produce a group decision while considering the opinions of others [1,2]. An essential component of this process is the need for individuals to adapt their opinions to others to reach a collective decision [3]. Determining the factors that underlie such judgment adaptation is, therefore, critical to understanding collective decisions. A key factor identified in social psychology that is known to influence whether subjects change their decisions to conform to those of others is group size [4,5]. Specifically, the larger the group size, the more people conform to the group’s opinion, but only up to a certain point [6,7]. To benefit from the opinion of others, the brain needs to track the likelihood that the group is making the most appropriate judgment [8] by estimating the credibility of social information [9–11]. One potential way to make such judgment adaptation to the group’s opinion possible is to assign greater credibility to a larger group’s opinion than to a smaller group’s opinion. Evidence for this mechanism come from studies comparing 6- and 12-person juries, showing that a larger jury is more likely to overcome its biases [12,13] and to obtain a result that represents the population mean more accurately than a smaller jury [14,15]. A statistical phenomenon that is known as the “wisdom of the crowd” also explains how the aggregated opinion of a group of individuals can be even more accurate than the estimates of experts [16–18]. Herding behavior in purchasing decisions [19,20] and collective animal behavior demonstrate a similar effect of group size on conformity [21,22]. These examples indicate the importance of group size for social decision-making. Assigning credibility to a more reliable source of information and reducing uncertainty are not only critical for learning appropriately but also to adapt to a group’s opinion [9,23]. However, little is known about how the brain estimates the credibility of aggregate opinions of groups of different sizes.

A mechanistic account of how the human brain integrates individual and social information for making decisions in groups is still unclear. Previous studies have established that people track and use social information based on its credibility [24,25]. However, so far, studies on social conformity have only accounted for the fact that changes in judgments within a group are driven by the motive to decrease social conflict [26–28]. In contrast, here, we considered that the changes in judgments in a group could be driven by belief updates that consider the credibility of each source of information rather than by social conformity. Such belief updates are distinct from social conformity, which exclusively concerns the difference between individual and social judgments. Moreover, previous studies did not take into account the confidence that people have in their own judgments and the influence of this confidence on judgment adaptation [11,29]. Yet, confidence in one’s decision plays a key role in revising one’s previous decision [30,31], and this is also likely to be true when integrating social information. Indeed, people are more likely to follow social information when they lack confidence in their own judgment [9]. Thus, we hypothesized that both confidence in one’s initial decision and group size are critical to account for integration of individual and social information during group decision-making.

**Fig 1:**
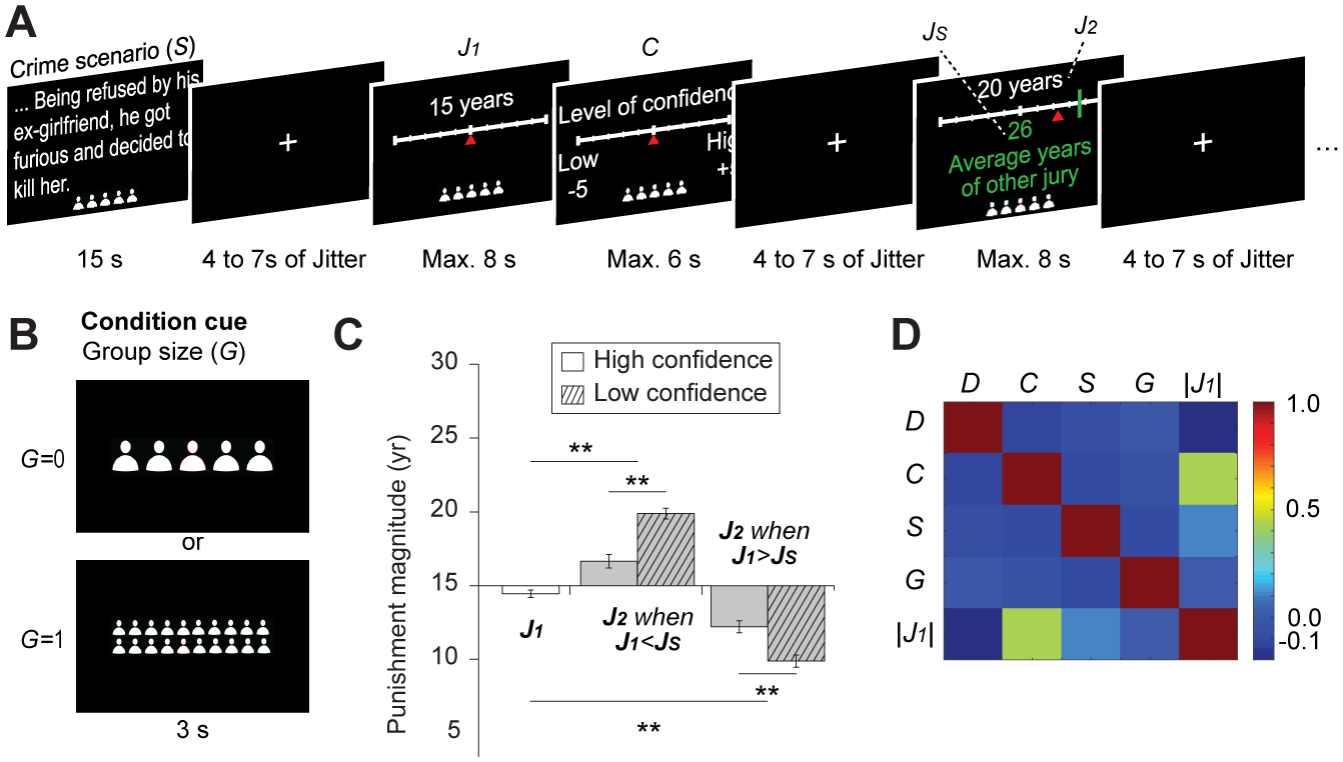
Experimental design and changes in legal judgments of participants while confronted with the judgments of other jurors. (**A**) After reading a murder case (52 scenarios in total), participants made their first judgment (*J*_*1*_) by deciding how many years in prison the criminal should be punished with, then they rated their own confidence level in this initial judgment, and eventually reconsidered their initial judgment to make their second and final judgment (*J*_*2*_) when provided with the averaged judgment of other jury members. Thus, participants could change the magnitude of their punishment from *J*_*1*_ to *J*_*2*_ after considering social information (the judgment of a social group, *J*_*S*_), which was the average number of prison years prescribed by the other jurors. Participants were informed that the other jurors were previous participants who had high-level confidence in their judgments. Our functional MRI (fMRI) analyses were focused on brain activity associated with judgment adaptation (6th screen) while controlling for other events. (**B**) A cue was shown at the beginning of each block to indicate the condition—5 jury members (small group trials, *G* = 0) or 20 jury members (large group trials, *G* = 1), providing the total number of members of the jury who participated in the judgment. (**C**) Behavioral results show that participants changed their initial judgment *J*_*1*_ to *J*_*2*_ to conform to the averaged judgment of other members, *J*_*S*_. Such change was modulated by the confidence that participants assigned to their initial judgment, *J*_*1*_ (**, *P* < 0.01). That is, in trials with low confidence, participants increased (respectively decreased) their sentence if it was lower (respectively higher) than the one from other jury members. (**D**) Color-map showing noncollinearity among factors considered to influence the changes in judgments. All experimental factors were independent, except for the correlation between one’s confidence level (*C*) and the initial judgments (*|J*_*1*_*|*) (*P* < 0.01). *D*: difference between judgments (*J*_*S*_ − *J*_*1*_); *S*: type of scenario (−1, sympathetic; 1, nonsympathetic scenarios). The data underlying Fig 1C and Fig 1D can be found in S1 Data.

Here, we examined the neural mechanisms enabling the consideration of the credibility of both individual information and social information. We first investigated whether the credibility of individual information and the credibility of social information are respectively modulated by one’s own confidence level and group size. Second, we investigated how the brain adapts to the judgments of others according to the credibility of each source of information. To address these questions, we tested different computational models that accounted for the changes in one’s judgments within a group as an adaptive decision-making process. Moreover, to characterize the neurocomputational mechanisms engaged in judgment adaptation, we used model-based functional MRI (fMRI) and a new paradigm in which participants were asked to make a series of punitive judgments on murder cases as part of a jury (Fig 1A). First, they made a judgment on the appropriate punishment (in prison years) for a criminal (first individual judgment [*J*_*1*_]; default was 15 years) along with an estimation of their level of confidence (*C*) in each judgment. They were then given the opportunity to reconsider and to update their first judgment (*J*_*1*_) to make a second judgment (*J*_*2*_) with the given knowledge of the average judgment of the other members of the jury (judgment of a social group [*J*_*S*_] where “S” stands for social information). Crucially, the size of the juries varied, being either large (20 jury members) or small (5 jury members) groups. Thus, the credibility of social information available to jurors could be estimated as either high or low, respectively (Fig 1B).

We developed and tested a Bayesian model of decision making under social influence. Neuroimaging studies of perceptual decision-making provide evidence that an observer has a mental model to conduct Bayesian inferences to infer probable states of the world from their observations [32,33]. In the field of social decision-making, although recent theoretical models proposed that such a Bayesian framework may be applied to understand decision-making under social influence [23,34], empirical evidence for characterizing the neural signals of such social decisions is still lacking. Our Bayesian model assumes that people process their own judgments and the judgments of others as distinct probability distributions considering the likelihoods of the most appropriate judgment. In this framework, the level of precision of each distribution represents the credibility that an individual assigns to each type of information [8,35,36]. We tested whether participants use, on one hand, the confidence levels in their own judgments to infer the credibility of their individual information and, on the other hand, the size of the group to estimate the credibility of social information. In such a Bayesian account, individuals integrate different sources of information and generate a novel belief [37], suggesting that the brain assigns different weights to individual and social information according to their respective credibility.

First, we compared our Bayesian model with alternative models. These alternative models had access to the same information about the choice options but assumed different computations when participants were presented with the judgments of others. We found that the Bayesian decision-making model explained the magnitudes of judgment changes of jurors better than other alternative models. That is, jurors integrated their individual levels of confidence and estimated levels of credibility of social information to construct a novel belief about what would be the most proper judgment and adjusted their judgments accordingly.

Second, we investigated the neural implementations of 2 fundamental computations of Bayesian inference during collective decisions, belief updates and credibility estimates of social information. We found that activity of the dorsal anterior cingulate cortex (dACC) reflected updates of one’s beliefs after integrating social information, rather than alternative computations from the other 2 models. For Bayesian computation, the belief update signals should monitor the cognitive process tracking changes in the credibility of social information. We thus further investigated which brain regions estimated the credibility of social information during judgment adaptation. To this end, we reasoned that if a region is engaged in computing the credibility of social information: (i) it should show greater activity when participants were given more-credible judgments by the large group than when given less-credible information by the small group; (ii) this region should provide greater inputs to the dACC to modulate one’s belief with increases in the credibility of social information, and this may be reflected by greater functional connectivity between this region and the dACC at the time of judgment adaptation; and (iii) both the activity and the functional connectivity of this region may represent individual differences in the willingness to assign greater credibility to judgments of the large group compared to judgments of the small group.

## Results

### Behavioral results

The mean punishment magnitude at the stage of the initial judgment (*J*_*1*_) was 14.44 ± 0.25 years (standard error of the mean [SEM]; Fig 1C, left graph). For trials in which the jury group was small, the mean judgment magnitude was 14.17 ± 0.35 years, and, for trials in which the jury group was large, the mean judgment magnitude was 14.71 ± 0.36 years. Magnitudes of initial punishment judgment did not differ between trials of different jury group size (*P* = 0.28). Participants changed their initial judgment, *J*_*1*_, to conform to the group opinion (*J*_*S*_) in 27.58 ± 1.47 trials on average. In the rest of the trials (24.42 ± 1.47), participants kept their initial judgments. We then investigated how the opinion of others influenced the second judgment (*J*_*2*_) by separating the cases in which the initial judgment was either lower or higher than the judgment of others. The range of the difference between the initial judgment and social information (*D* = *J*_*S*_ − *J*_*1*_) was, by design, divided into 2 intervals of punishment years, and the group never had the same level of punishment judgments as individual participants (*J*_*1*_ ≠ *J*_*S*_; see Materials and methods). In addition, the mean confidence rating was 0.69 ± 0.22, being normalized on a scale from 0, no confidence at all, to 1, high confidence. We confirmed that all participants chose the lowest and the highest confidence rating at least once. Confidence did not differ between the size of jury group (F = 4.91, *P* = 0.27) or between blocks (F = 3.81, *P* = 0.34).

We observed that individuals tended to increase the magnitude of their punishments (*J*_*2*_ = 18.93 ± 0.33 years, *P* < 0.001) when the judgments of group were more severe than those of the juror (*J*_*S*_ > *J*_*1*_) and tended to decrease the magnitude of their punishments (*J*_*2*_ = 10.82 ± 0.30 years, *P* < 0.001) when the judgments of group were more lenient than those of the juror (*J*_*S*_ < *J*_*1*_). To investigate whether changes in judgments (*J*_*2*_ − *J*_*1*_) were driven by the perceived difference in judgments (*J*_*1*_ − *J*_*S*_), we computed the level of conformity (*LC*; Eq 1). Based on this measure, conformity trials were defined as *LC* > 0 and nonconformity trials as *LC* ≤ 0. The average level of conformity was strictly positive (*LC* = 0.24 ± 0.01), suggesting that the effect of social influence was significant.

Next, we investigated how different levels of confidence assigned to the initial judgments (*J*_*1*_) influenced the level of changes in judgments. After median splits of all trials based on each individual’s confidence ratings, we observed that confidence had a similar effect on changes in judgments (from *J*_*1*_ to *J*_*2*_). That is, on average, people tended to conform more to the group opinion when they had low confidence in their initial judgment. When one’s initial judgment was more lenient than the judgment of the group, individuals tended to increase the magnitude of their punishments more when they were less confident of their initial judgment (*J*_*2*_*L* (low confidence) = 19.88 ± 0.36 years, *P* < 0.001) compared to when they were more confident of their initial judgment (*J*_*2*_*H* (high confidence) = 16.64 ± 0.46, *P* < 0.001, Fig 1C, middle graphs). When the initial judgment was more severe than the judgment of the group, individuals tended to decrease the magnitude of their punishments more when they were less confident of this initial judgment (*J*_*2*_*L* = 9.88 ± 0.41, *P* < 0.001) compared to when they were more confident of this initial judgment (*J*_*2*_*H* = 12.21 ± 0.42, *P* < 0.001; Fig 1C, right graph).

To clarify how the factors manipulated in the task influence changes in judgments (*J*_*2*_ − *J*_*1*_), we performed a statistical analysis using the linear mixed-effects modeling analysis (LMEM). We found that changes in judgments significantly depended on the difference between judgments (*D*, F = 31.79, *P* < 0.01), their confidence levels (*C*, F = 2.67, *P* = 0.003), their interaction (*D × C*, F = 2.45, *P* < 0.01), and the interaction between the difference in judgments and group size (*D × G*, F = 2.05, *P* = 0.02). These changes did not depend on the group size (*G*, F = 3.49, P = 0.12), the types of scenario (*S*, F = 1.05, *P* = 0.39), the regression-to-the-mean effect of the initial judgments (|*J*_*1*_|, F = 3.12, *P* = 0.12; see Materials and methods for details), and the interaction between the difference between judgments and types of scenario *D × S* (F = 1.19, *P* = 0.28). To illustrate this effect more clearly, we performed additional factorial analyses. By splitting all trials with high- and low-confident judgments using each individual’s median value of confidence rating, we found that participants were more likely to conform to others when confidence in their judgments was low (F = 61.64, *P <* 0.001), which provided additional evidence supporting the effects of confidence level (*C*) on decisions to conform to the judgments of others (*LC*).

We further examined whether some of these experimental factors are correlated with each other. The level of collinearity between experimental factors is shown in Fig 1D. As illustrated, we only found a relationship between initial judgments (|*J*_*1*_|) and level of confidence (*C*). That is, participants tended to make more extreme judgments (close to 0 or 30 years) when they had greater confidence in their judgments (R^2^ = 0.43, *P* < 0.01). This correlation might be caused by the “status quo”—the tendency of decision-makers to stay close to the given default when having low confidence in their decision [38,39].

### Computational modeling of behavioral data

To examine how the brain computes social updating during group decision-making and to know whether and how much the brain decides to change initial judgments, we proposed and compared 3 computational models.

The “linear model” (Eq 7) predicts changes in judgments by a linear regression model that takes into account one’s initial judgments (*J*_*1*_), given deviation in the group judgments (*D* = J_S_−J_1_), the level of confidence that one had for their judgments (*C*), and 3 interaction effects considering group size (*G*) and the types of scenarios (*S*) (*D × C*, *D × G* and *D × S*). In doing so, the “linear model” tests whether individuals are motivated to reduce the perceived deviation in the group judgments by adjusting their prior judgments to be more consistent with that of the group.

The “surprise model” (Eq 8) assumes that participants change their judgments more when they are more likely to observe unexpected group judgments, given their prior belief about the most preferable judgments (*J*). Thus, surprising events have greater impacts to drive changes in judgments. According to information theory [40], surprise evoked by the observation of unpredicted event *α* is computed as the inverse entropy of the predictability of the event, participants having their own prior belief (−*log p*(*α*|*Prior*)). The level of precision of the prior belief, thus, was computed as the variance of a normal distribution, which depends upon a function of confidence ratings, (*p*(*J*_1_|*Prior*) ~ *N*(*μ* = *J*_1_, *σ*^2^ = *f*(*C*)^−1^). Moreover, we assumed that participants were more surprised when the deviation (*D*) was given from the judgments of a larger group (*D × G*) and when the group proposed a more severe (or more lenient) level of punishment than their own when making judgments for the sympathetic (or nonsympathetic) scenarios (*D × S*).

**Fig 2:**
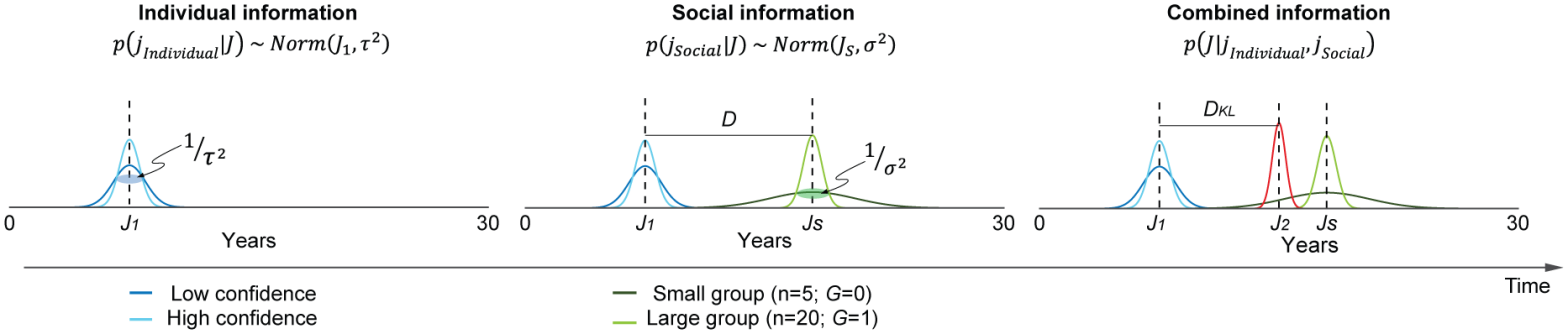
The Bayesian model predicting the changes in judgments under social influence. Individuals form a private estimate of the most preferable level of punishment for a criminal and make their initial judgments (*J*_*1*_). When the judgments of other social group members (*J*_*S*_) are observed, participants assigned weights to individual (*J*_*1*_; left panel) and social information (*J*_*S*_; middle panel) according to their respective credibility. These 2 beliefs are integrated into the final estimate (*J*_*2*_; right panel). Judgments are adjusted according to this final estimate. Changes in judgments from *J*_*1*_ to *J*_*2*_ were predicted by the belief updates, estimated by Kullback–Leibler divergence (*D*_*KL*_).

The “Bayesian model” (Eq 6) assumes that participants tried to estimate the most preferable judgments (*J*). The punishment—the years of prison that the defendant deserved—is dependent on this estimated judgment. Initial judgments (*J*_*1*_) were based on individuals’ private estimates (*j*_*Individual*_) after reading the scenario of a crime case. The distribution, *p*(*j*_*Individual*_|*J*), represented the probability that their private estimate was the most preferable. The credibility of individual information, thus, was represented as the level of precision of a normal distribution that depends on a function of confidence ratings, *p*(*j*_*Individual*_|*J*) ~ *N*(*μ* = *J*_1_, *σ*^2^ = *f*(*C*)^−1^). When social information is presented, participants may assign different levels of credibility as a function of changes in group size, *p*(*j*_*Social*_|*J*) ~ *N*(*μ* = *J*_*S*_, *σ*^2^ = *f*(*G*)^−1^). More importantly, to explain that changes in judgments are driven by changes in one’s belief about what would be morally correct and what level of punishment would be the right amount for the crime, the “Bayesian model” integrates available information while considering their level of credibility. Therefore, this model also assumes that the brain computes the extent of belief updates. The extent of belief updates was well captured with Kullback–Leibler divergence (*D*_*KL*_), which shows the dissimilarity between 2 probability distributions of the individual estimate, *p*(*j*_*Individual*_|*J*), and final estimate, *p*(*J*|*j*_*Individual*_, *j*_*Social*_), of individuals (Fig 2).

### Parameter estimation

**Fig 3:**
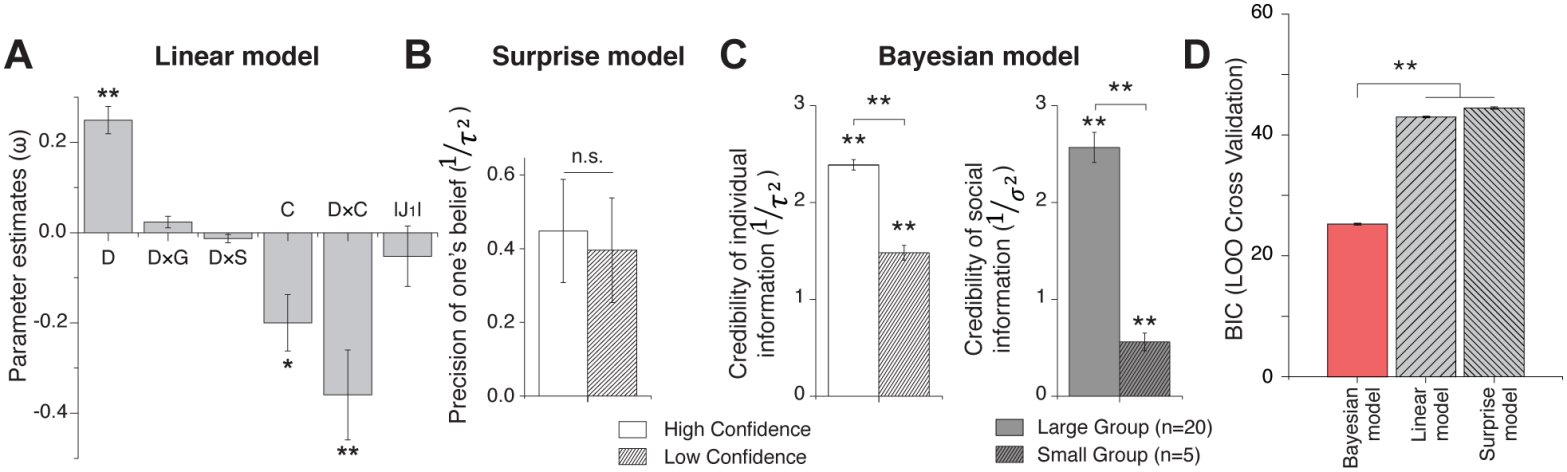
Model comparisons of the predictability of the changes in judgments. (**A**) Parameter estimates in the “linear model” indicating that participants assign a significant weight to the differences in judgments (*D*), their confidence level (*C*), and the interaction between them (*D × C*), but not to the interaction with group size (*D × G*) and scenario types (*D × S*) (Eq 7). Error bars represent the standard error of the mean (SEM). (**B**) The “surprise model” hypothesized that participants who have greater confidence in their judgments are more likely to be surprised by unexpected differences in judgments with a social group and to adapt their judgments to the given judgments of groups. However, the parameter estimates in this model suggest that the different level of confidence could not explain the different level of judgment adaptation (Eq 8). (**C**) The “Bayesian model” (Eq 6) provides 2 individual, specific functions that allow us to track the changes in credibility. First, individual jurors were more likely to assign credibility to their individual information (*J*_*1*_) when they had a higher level of confidence (left panel). Second, participants assigned more credibility on social information (*J*_*S*_) when it was provided by a larger (*n* = 20) compared to a smaller (*n* = 5) group (**, *P* < 0.001; right panel). **(D)** The likelihoods of each model prediction were estimated by a leave-one-out-cross-validation (LOOCV) procedure, and the Bayesian information criteria (BIC) were compared between models. The BIC showed that predicted changes in judgments of the “Bayesian model” fit better to the actual changes in judgments than the other models (**, *P* < 0.001). The data underlying this figure can be found in S1 Data.

The parameters that maximize the likelihood of the model based prediction of the actual changes in judgments were estimated for each of the 3 computational models. The parameter estimates in the “linear model” suggest that changes in judgments were significantly driven by the difference in judgments (*D*), the level of confidence in the initial judgments (*C*), and their interaction (*D × C*) (*P* < 0.05, 1-sample *t* test, Fig 3A).

Notably, both the “surprise model” and the “Bayesian model” assume that the confidence level in one’s own belief serves to estimate the level of precision of the prior belief. In contrast to the “Bayesian model,” in which greater credibility is assigned to more confident judgments, the “surprise model” assumes that participants are more likely to be surprised by the unexpected judgments of others, especially when they had a high degree of confidence in their own judgment [33,40]. However, the parameter estimates showed that the “surprise model” could not capture the different levels of judgment adaptation when confidence varied (Fig 3B).

The “Bayesian model” not only took into account the punishment magnitude of individual judgments, *J*_*1*_, and the judgments of others, *J*_*S*_, but also the beliefs that the participant had about the credibility of individual information and social information, respectively. This framework allowed us to find the credibility that participants assigned to their individual information as a function of confidence rating and its updates after integrating social information according to its credibility. The relationship between confidence reports and the credibility of individual information was measured by a parameter, *ω*_*C*_, across participants (Eq 2). The mean parameter estimate of *ω*_*C*_ was 1.13 ± 0.47, which was significantly greater than 0 (*P* < 0.001, 1-sample test), suggesting its significant effect. That is, participants put more credibility on their own judgments when they had higher confidence (F = 15.63; *P* < 0.001; LMEM; Fig 3C, left graph). Moreover, we tested whether participants took into account changes in group size when estimating the credibility of social information through the integration of social information with their prior individual information. The “Bayesian model” provided evidence of significant effects of group size on the credibility of social information. Again, we observed that participants assigned more credibility to the judgments of a larger group (F = 121.77; *P* < 0.001; 1-way ANOVA; Fig 3C right graph).

### Comparisons between Bayesian model and alternative models

Among the models, the Bayesian model offered a better fit than the others (comparing the likelihood of each model, *P* < 0.001, 1-way ANOVA; Eq 10). The mean –2log likelihood of the Bayesian model was 8.55 ± 0.16, while those of the “linear model” and the “surprise model” were 14.12 ± 0.25 and 20.62 ± 0.44, respectively. To avoid potential overfitting, we predicted each of the levels of judgment adaptation across trials of all participants using an iterative leave-one-out-cross-validation (LOOCV) procedure and estimated the likelihood of the prediction and actual changes in judgments. Furthermore, we penalized the likelihood and computed the Bayesian information criteria (BIC) that took into account the number of free parameters in each model (*P* < 0.001, 1-way ANOVA, Eq 11; Fig 3D).

### Neural networks underlying Bayesian computation of judgment adaptation

**Fig 4:**
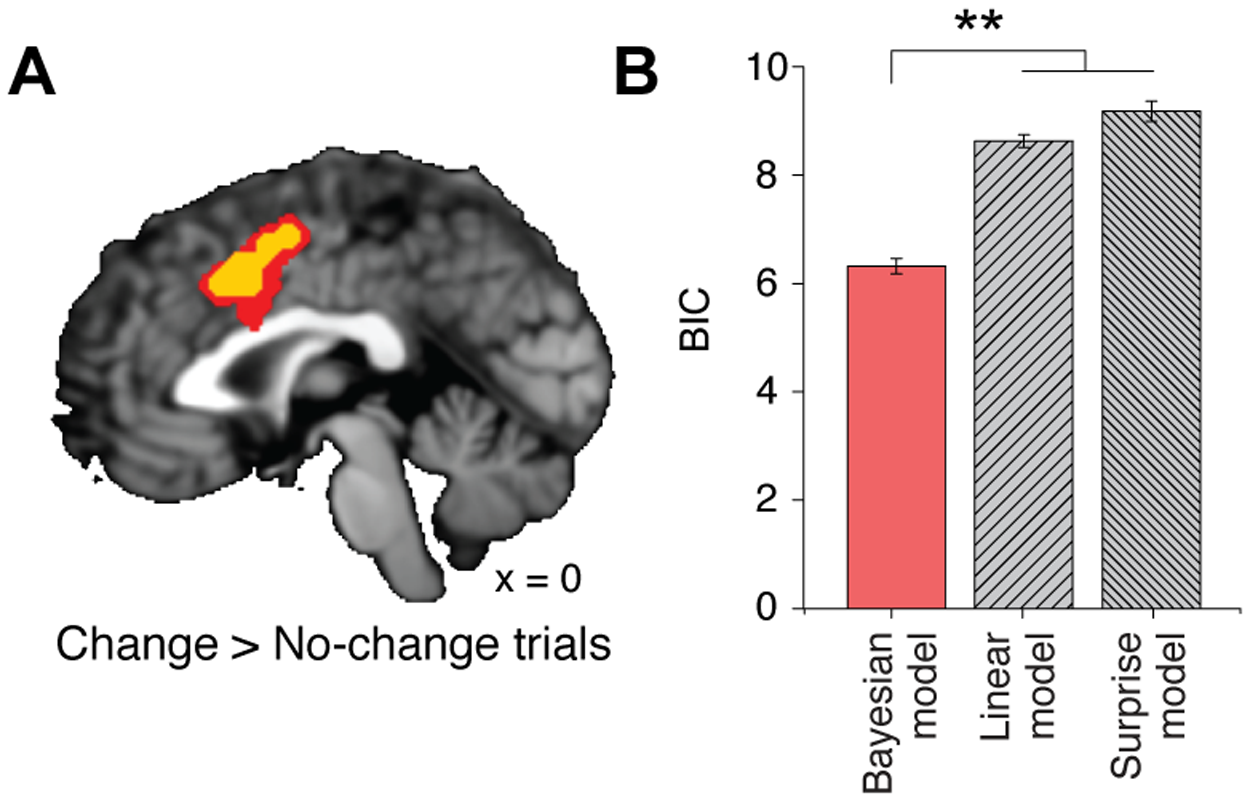
Dorsal anterior cingulate cortex (dACC) activity when changing judgments to conform to the group’s and comparison between the predictions of different models in this brain region. **(A)** The dACC was more engaged with conformity trials compared with nonconformity trials. (**B**) The Bayesian model explains dACC activity more accurately than those of alternative models (general linear model [GLM]3). The time courses accounting for the blood-oxygen-level dependent (BOLD) signal in association with the changes in judgments were extracted from the region of interest (ROI) in dACC. These time courses were compared with other time courses in association with the predictions of each model about changes in judgments. Error bars represent the standard error of the mean (SEM). All the parameters were estimated from the same ROI defined in the dACC (10-mm diameter spherical ROI at x, y, z = 8, 18, 46; **, *P* < 0.001). The functional MRI (fMRI) data can be found in http://neurovault.org/collections/2503/. The data underlying Fig 4B can be found in S1 Data.

We found that dACC activation (peak voxel x,y,z = −3,14,44) increased when participants reconsidered their initial judgment and adapted it towards the judgment of the group (P < 0.05, family-wise error [FWE] corrected within small volume correction [SVC], T = 4.45, Fig 4A). We also found dACC activation in the same cluster (peak voxel x,y,z = 0,12,39) when an alternative general linear model (GLM) with a fixed boxcar duration independent of reactions times (= 4 s) was applied (*P* < 0.05, FWE corrected within SVC, T = 4.75; S1 Fig). This alternative model allowed us to exclude alternative hypotheses regarding dACC functions as reflecting speed of response or invigoration rather than judgment adaptation.

Having established that the Bayesian model better accounted for the behavioral data than other models, we tested whether the changes in judgment predicted by the Bayesian model more accurately explain the conformity-related brain signal in dACC than those predicted by the other models. To address this, we used an additional GLM (GLM3) in which the brain response was modeled by 4 parametric regressors without serial orthogonalization. We then extracted the time courses of beta parameters from the dACC ROI. The goodness-of-fits of those time courses were measured with the likelihood of predicting the dACC time courses that represented the changes in judgment. The mean value of −2log likelihood and BIC (in parenthesis) are as follows: 2.37 ± 0.14 (6.32 ± 0.14) for the Bayesian model, 4.68 ± 0.12 (8.63 ± 0.12) for the “linear model,” and 5.23 ± 0.19 (9.18 ± 0.19) for the “surprise model.” In addition, we computed BIC to penalize likelihoods by the numbers of free parameters used by each model. We found that the time courses extracted from the “Bayesian model” explained conformity decision–related dACC activity more robustly than time courses extracted from the other models (*P* < 0.001, 1-way ANOVA, Fig 4B).

We also performed the same analysis with an alternative GLM using a fixed boxcar duration of 4 s. The time courses of beta parameters were extracted from the same predefined dACC ROI. The goodness of fit of those time courses of model predictions was measured with the likelihood and BIC. The mean value of −2log likelihood and BIC (in parenthesis) are as follows: 2.69 ± 0.20 (6.64 ± 0.20) for the Bayesian model, 5.16 ± 0.11 (9.11 ± 0.11) for the “linear model,” and 5.80 ± 0.17 (9.75 ± 0.17) for the “surprise mode.” We found that the Bayesian model was significantly lower than those of other models (*P* < 0.001, 1-way ANOVA).

To ensure that dACC activation cannot be explained by response selection difficulty, as suggested previously by Shenhav and colleagues [41–43], we performed a similar analysis to the one from O’Reilly and Kolling [33,44,45] by including, in the same GLM, the update term regressor *D*_*KL*_ as well as the level of surprise and reaction times (GLM4). This allowed the belief update regressor to compete with these measures of difficulty to explain variance in dACC activity. The results of this new GLM confirmed the robustness of our dACC activity as reflecting a Bayesian update signal of one’s beliefs after integrating social information (peak voxel x,y,z = 6,8,49; *P* < 0.05, FWE corrected within SVC; S2 Fig). This result demonstrates that this dACC signal can be dissociated from tracking choice difficulty.

Computationally, tracking changes in the credibility of social information is required to update one’s belief. If dACC activity represents the level of belief updates as evidenced by the results of the “Bayesian model,” then this brain region would require feedbacks when the credibility of group judgments changes. Moreover, if the credibility of group judgment is processed separately by other brain areas, functional connectivity to dACC should increase in order to guide subsequent judgment adaptation. We thus tested for brain regions implicated in social credibility during the integration of social information.

**Fig 5:**
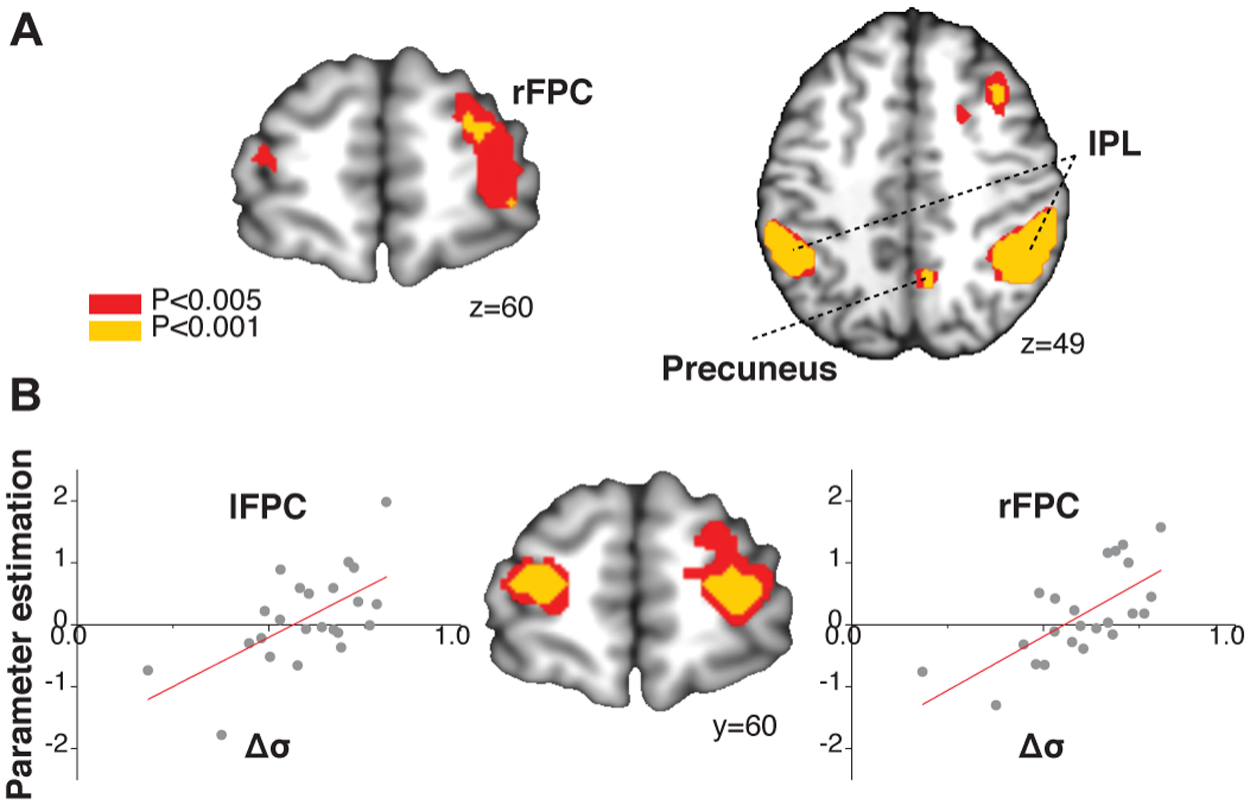
Brain regions engaged in social credibility while integrating social information and individual information to update one’s belief about the preferable judgments. (**A**) The activity in the right lateral frontopolar cortex (FPC; [x,y,z] = [42,44,19]), in the precuneus (x,y,z) = (18,−58,31), and in the bilateral inferior parietal lobule (x,y,z) = (60,−34.43) and (−54,−49,52) was greater when participants were given the high-credible judgments of the large group than those when they were given the relatively low-credible information of the small group (*P* < 0.05, whole-brain family-wise error [FWE] corrected at cluster level; general linear model [GLM]2). (**B**) Whole-brain regression analysis between brain activity and the individual differences, *Δσ*, represents the ratio between the credibility of social information in the large group and the credibility of social information in the small group. We found that the different activity in the bilateral FPC represented the individual differences in *Δσ*. That is, participants with greater activation in the FPC showed higher differences in the credibility that they assigned to the judgments of a larger group relative to those of a smaller group (P < 0.05 FWE corrected at the cluster level; GLM2). The functional MRI (fMRI) data can be found in http://neurovault.org/collections/2503/. The data underlying Fig 5B can be found in S1 Data.

First, we found that activity in the right lateral frontopolar cortex (FPC; [x,y,z] = [42,44,19]), in the precuneus (x,y,z) = (18,−58,31), and in the bilateral inferior parietal lobule ([x,y,z] = [60,−34.43] for right inferior parietal lobule [iPL] and [−54,−49,52] for left iPL) was greater when participants were presented with the judgments of large group compared to when presented with the judgments of small group (Fig 5A; *P*_FWE_ < 0.05, whole-brain corrected at cluster level; GLM2).

Second, to investigate which brain regions show individual differences in relative credibility as a function of group size, we performed a whole-brain regression analysis with *Δσ* (Eq 4). We found that the bilateral FPC was the only area representing this parameter: left FPC peak voxel (x,y,z) = (−30,58,15); right FPC peak voxel (x,y,z) = (27,57,10) (Fig 5B; P_FWE_ < 0.05; whole-brain corrected at the cluster level; GLM2). This result indicates that participants who had greater activation in the FPC showed higher differences in the credibility that they assigned to the judgments of a larger group relative to those of a smaller group. By extracting the parameter estimates from FPC activations, we showed a linear relationship between individual differences in FPC activation and sensitivity to group sizes for assigning credibility to social information. Moreover, these effects were significant when we applied bootstrapping sampling: the effect size in right FPC was 3.53 ± 0.18 (SEM) with a 95% confidence interval between 2.16 and 5.53; the effect size in left FPC was 3.11 ± 0.24 (SEM) with a 95% confidence interval between 1.22 and 5.68 (*P* < 0.001). Moreover, to further check this effect, we also tested whether any brain activity represented the scrambled individual parameter (Δσ), in which we randomly assigned each participant’s Δσ to another participant. We found no significant neural correlates of the scrambled Δσ even at a liberal statistical threshold (*P* < 0.005, uncorrected).

Third, given that results indicate that only the right lateral FPC satisfies both criteria, we further tested whether functional connectivity between the dACC and right lateral FPC was modulated by the size of the group while making decisions of judgment adaptation.

To do this, we performed psychophysiological interaction (PPI) analyses. In the PPI analysis, we took as a seed the right lateral FPC and tested whether its functional connectivity to the dACC was modulated during judgment adaptation. The ROI was defined as a 10-mm diameter spherical ROI at the peak voxel in the right lateral FPC, (x,y,z) = (42,44,19), which was identified as being modulated by group size (large group trials > small group trials; GLM2). We computed PPI maps to identify the brain areas for which functional connectivity with the right FPC increased in trials in which participants were confronted with judgments of the large group compared with trials in which they were confronted with judgments of the small group.

**Fig 6:**
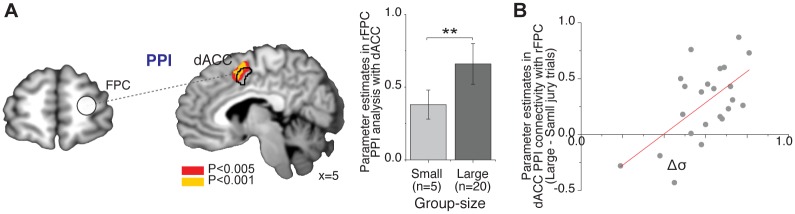
Functional connectivity between lateral frontopolar cortex (FPC) and dorsal anterior cingulate cortex (dACC). (**A**) Psychophysiological interactions (PPI) analyses. The PPI used the right lateral FPC as a seed (sphere of diameter = 10 mm centered at [x,y,z] = [42,44,19]). The functional connectivity between right FPC and dACC increased when group size increased (*P* < 0.05, family-wise error [FWE] corrected within small volume correction [SVC]). Error bars represent the standard error of the mean (SEM) (**, *P* < 0.001). (**B**) Between-subject regression analysis considering the individual difference in the relative credibility of social information (*Δσ*) as a covariate for the modulation of connectivity between dACC-FPC when presented with social information of the large versus the small group (*P* < 0.05, FWE corrected within SVC). The scatter plot was not used for statistical inference (which is carried out in the SPM framework). It is shown solely for illustrative purposes. The functional MRI (fMRI) data can be found in http://neurovault.org/collections/2503/. The data underlying this figure can be found in S1 Data.

We found that changes in group size modulated connectivity between dACC and right lateral FPC (P_SVC_ < 0.05, FWE corrected within the small volume cluster in the dACC ROI, peak [x,y,z] = [9,11,43]; Fig 6A). We also confirmed that the dACC region for which functional connectivity to the lateral FPC was modulated by group size is located in the same dACC cluster as that observed for conformity decisions (Conformity > No-conformity; black contour in Fig 6A).

We further found that individual variability in the strength of functional connectivity between large group (*n* = 20) trials and small group (*n* = 5) trials correlated with Δσ, the tendency of individuals to assign greater credibility to the group’s opinion when the group size was large (Fig 6B). These results showed that connectivity between the FPC and the dACC increased in those individuals with higher sensitivity to group size. Thus, both FPC activity and the strength of FPC-dACC coupling influenced the degree to which social information was integrated into the posterior belief of individual participants.

## Discussion

Our findings show that a Bayesian model provides a good account of observed behavior when a judgment based on a private estimate (individual information) is confronted by the aggregated opinion of fellow members of a group (social information). According to our Bayesian model, participants used both the confidence in one’s own judgments and group size of social information to estimate the credibility of each type of information. Participants thus weighed their initial judgment and the judgment of others by their respective level of credibility, integrated them into a new belief, and changed their punitive judgments accordingly. Our Bayesian model explains judgment-adaptation behavior better than other models. The Bayesian model differs from alternative models in an important way. It predicts that judgment adaptation should be sensitive to the credibility of both individual and social information, whereas other models predict exclusive sensitivity to the level of social conflict.

When individuals made a decision to change their initial judgments to fit in with that of the group, we observed that participants did not simply conform more to a larger group than to a smaller group, nor did they have a higher level of social conflicts with the judgments of a larger than a smaller group. Instead, participants tended to attribute more credibility to a larger than a smaller group. Only the Bayesian model captures this effect. These results explain a long-standing debate about the relationship between group size and social conformity [6]. While some studies have observed that increasing group size does not influence conformity to the group beyond a minimal number [4,5], others have reported that the larger the size of the group, the larger the effect [46]. These studies suggest that the relationship between conformity and group size cannot be described by a simple function but instead varies systematically with factors that impact social-influence processes.

At a mechanistic level, our Bayesian model describes the neurocomputational mechanisms underlying judgment adaptation in a group. A number of frontal cortex regions have previously been shown to be engaged during social decision-making, such as the dACC and the FPC. Here, we show that specific signals integrating individual and social information are computed in these 2 regions and shared between them. In particular, we investigated the neural implementations of 2 fundamental computations of Bayesian inference during collective decisions: belief updates and credibility estimates of social information. The dACC computed the belief updates that were necessary to adapt judgments, while the FPC computations reflected the credibility that people assigned to social information. Furthermore, an increase in functional connectivity between these 2 regions predicted individual differences in credibility assigned to the judgments of a larger group, compared to the one assigned to the judgments of a smaller group. The estimate of credibility of social information, computed in the FPC, was critical for efficient Bayesian computation of belief updates, processed in the dACC. By monitoring the changes in the credibility of social information, the FPC may test the validity of individual information and modulate the integration of social information.

Our results suggest a general functional role for the dACC, which is to update one’s belief by integrating different types of information according to their respective credibility. Several accounts have proposed that the dACC is engaged during conflict monitoring [45,47], and this general function has been recently extended to the social domain, possibly reflecting computation of the difference between one’s own judgment and those of others (“social conflict” hypothesis) [26,48,49]. However, if conformity is only understood as a resolution of social conflicts, the changes in judgments should exclusively depend on perceived differences in judgment (*D = J*_*S*_*−J*_*1*_) but not on the changes in credibility of each source of information. Thus, the effects of changes in confidence (*C*), group size (*G*), and their interaction effects with the perceived differences in judgments (*D* × *C* and *D* × *G*) should be independent from the changes in judgments. Here, by incorporating the changes in credibility of each source information, however, we provide evidence that the Bayesian model captures the variances of judgment adaptation across trials. Specifically, in some instances, individuals maintain their initial judgments, while in other instances, the same individuals change their judgments to conform to the group, even though they are faced with the same level of social conflict. We found that participants with high confidence in their judgments tended to assign greater credibility to individual information. Moreover, the Bayesian model shows that the dACC encodes the updates of the beliefs about the validity of one’s judgment (*D*_*KL*_) after integrating the judgments of others. This Bayesian model allows us to explain intra-individual variability in judgment adaptation and to account for dACC activity better than other psychological conformity models. A previous study using a saccadic planning task demonstrated that the dorsal anterior cingulate cortex (dACC) was activated when updating internal models about the probabilistic state of uncertain environments while integrating perceptual information [33]. In the current study, we found that belief updates about the best judgment to make also rely upon dACC computations. That is, the activity of the dACC reflected updates of one’s beliefs after integrating individual information with social information, rather than alternative computations from the other 3 models. By providing evidence that the dACC contributes to belief updates in the context of our group decision-making task, our findings generalize this computational role of dACC to the domain of social decision-making. However, we do not claim that this region is engaged in processing social information per se. In fact, other regions of the ACC appear to have much more specialized roles in social cognition [50–52]. In particular, the anterior ACC gyrus may be more engaged in tracking the intention of others or in computing the costs and benefits of acting in social contexts, whereas the ACC sulcus may be more involved in monitoring the value of one’s own action [53,54].

It could be argued that dACC activation reflects increases in decision difficulty rather than model updating [41,43]. According to this interpretation, dACC signals the need for control when overriding a default belief. In the Bayesian model, the optimal decision of when to update one’s belief depends on when the value of a conformity decision is equal to the value of keeping the previous decision. In many decision-making situations, decision difficulty and belief updates are confounded. Critically, in our study, decision difficulty can be distinguished from belief updates when participants had low levels of confidence in their individual information and were confronted with large differences in social information. In such cases, participants needed to update their belief by a large amount, but at the same time, the decision was easy. If dACC activity represented decision difficulty rather than belief updates, the Bayesian model would not be able to explain the neural correlates of judgment changes better than other models. Indeed, we found that the dACC activity predicting conformity decisions is explained by Bayesian belief updates measures (referred to as *D*_*KL*_), even in a GLM allowing belief updates to compete with measures of difficulty (level of surprise and reaction times) to explain variance in dACC activity [33,44,45]. In addition, if greater surprise caused the larger demands for control (corresponding to decision difficulty), then dACC activity would have been better explained by the surprise model, which was not the case.

In addition, our findings demonstrate that the FPC computes the credibility that people assign to social information. That is, those individuals who assign a greater credibility to social information with larger group sizes show higher activity in FPC when integrating the social information of a larger group compared to that of a smaller group. Moreover, our neuroimaging results indicate that FPC activity, and also FPC-dACC connectivity, play an important role in regulating the degree to which group size influenced credibility estimates of social information. Indeed, both FPC activity and the strength of connectivity between the dACC and FPC predicted inter-individual variances in the credibility assignment to the judgments of a group as a function of changes in group size. For adaptive decision-making in ever-changing environments, the human FPC has been reported to serve probabilistic inferences about the credibility of available information to make optimal use for decision-making [55–57]. Moreover, the lateral FPC is involved in monitoring alternative behavioral strategies and in deciding to switch to alternative courses of actions when one alternative strategy becomes more credible in comparison with the ongoing one [25,58,60]. Activity in this region also reflects individual differences in the extent to which learning is driven by the prediction errors of one’s belief about the best judgments when new evidence is given [59]. Consistent with this role, our results provide evidence that the FPC processes the credibility of alternative opinions. Thus, a key role of the FPC in social decision-making may be to monitor the credibility of social information when individual information is uncertain. This is an essential capacity to promote flexible behavior in environments in which the current decision strategies become unreliable.

It is worth noting that the FPC may have evolved to manage our unusually complex social systems [60,61]. In nonhuman primates, gray matter density of the FPC has been reported to increase with social network size [62]. The FPC, which develops late both from a phylogenetic and ontogenetic perspective in humans, may serve demands requiring interactions with larger social groups. Such cognitive demands of sociality could place a constraint on the number of individuals with whom we can interact and maintain contact with [60,62].

The current findings differ from previous studies on social influence and conformity [49,63] by allowing us to dissociate the credibility of social information from changes in confidence/uncertainty in one’s belief. These previous studies reported ventromedial prefrontal cortex (vmPFC) activation of choice options that vary with the choices of others [49], as well as with the level of confidence in the choices of others [63]. Such vmPFC activity may signal increases in the confidence of individuals’ decisions once social information has been integrated with individual information, reflecting the possibility that participants gain reassurance in their choice from the choices of others who are confident in their decision. This interpretation is supported by the recent findings showing that vmPFC activity encodes decision confidence by reflecting the amount of accumulated evidence favoring one option over the others [38,64]. Future work will need to measure the level of confidence not only after making the initial judgment but also after the judgment adaptation to clarify how vmPFC, dACC, and lateral FPC interact to flexibly exchange information at these different stages of social decision-making.

While most previous research has focused on ACC or FPC functions in isolation [45,65], dACC-FPC interactions have been relatively unexplored. Our results show that a higher degree of credibility to a larger group was reflected by an increase in the connectivity between the dACC and FPC. This change in the strength of the functional connectivity between the dACC and FPC may reflect a readout function of the dACC from the FPC to compute the Bayesian inference, since credibility estimates of social information are required to update one’s belief in an optimal fashion. Thus, our findings suggest the dACC-FPC network plays a key role in the context of group decision-making and judgment’s adaptation in the context of social decision-making.

To develop neurocomputational models of social decision-making, we specified which variables are computed for judgment adaptation within a group and how they are implemented in specific brain regions. A key novel aspect of the current study is to determine the behavioral algorithm for belief updates of individuals about the most preferable judgment and examine the neural correlates of this process. This is distinct from past studies asking participants to rate their preferences for goods or attractiveness of faces [26–28], in which the credibility of different sources of information was not relevant to make a decision of social conformity. The crucial novel contributions of the present study are also as follows: (i) to determine how the credibility of individual and social information are computed for updating one’s judgment, taking into consideration one’s confidence and group size, which was ignored in previous preference-based rating paradigms [26,27,66]; and (ii) to test a Bayesian model against plausible alternative models, which were matched with regards to access to information about the choice options.

Together, our findings transform the current thinking about the neural basis of conformity and collective decision-making by proposing a neurocomputational understanding about how individuals adapt their judgments by integrating social influences of other individuals in a group. Our study specifies how the human brain benefits from the wisdom of a larger group while preserving confidence in one’s initial judgment [10]. It also delineates the neurocomputational mechanisms at the source of inter-individual differences in assigning credibility to the opinion of groups with different sizes. By identifing the brain region tracking the credibility of social information, our findings also provide a mechanistic account of the computational mechanisms underlying judgment adaptation during collective decision-making.

## Materials and methods

### Ethics statement

This study was approved by the Institutional Review Board of the local ethics committee (Lyon, France, IRB n°A13-37030), and all participants gave their informed written consent.

### Participants

Participants were 25 healthy French volunteers (age range 20–26 years, 13 males). Data from 2 participants were discarded because of excessive movements during scanning. Therefore, data from 23 subjects (11 males; mean age 21.22 ± 0.463 years; error indicates SEM) were included in the final analysis.

### Task design

During this experiment, participants were asked to make a series of punitive judgments on murder cases as part of a jury while undergoing conditional blocks of fMRI. At the beginning of each block, we displayed the size of the jury for 3 s (either 5- or 20-person juries) to inform participants of how many individuals were also making a judgment in the current murder case along with them. In each trial (60 trials in total), subjects first read the scenario of a murder case for 15 s. They then made 3 successive decisions for each murder case.

First, participants were asked to make a judgment about how many years of prison the defendant deserved (*J*_*1*_). They reported it by moving a continuous numerical sliding cursor from the initial sentence of 15 years to the final value of prison years ranging from 0 to 30 for 8 s. We also informed participants that 15 years is the average prison sentence for murder case trials in France. The trials for which the participants failed to respond within 8 s were excluded from the further analyses. The position of the cursor was marked on the screen with the corresponding number of years in prison to make sure that the participants were aware of their judgment.

Second, participants had 6 s to rate the level of subjective confidence (*C*) that they had while making their *J*_*1*_, using a Likert scale of 10 items from −5 (low confidence) to +5 (high confidence) by moving a similar cursor on the screen.

Third, participants were given a chance to reconsider their first judgment (*J*_*1*_) with the knowledge of the average sentence given by the other members of the jury (*J*_*S*_). Social information was revealed by a green bar on the slider with its corresponding numerical value in prison years (bottom of the screen). During 8 s, the participants could review *J*_*1*_ by moving the cursor from the value they had chosen during their first assessment to one corresponding to their desirable reconsidered judgment (*J*_*2*_).

To encourage fully sincere judgments of participants who may moderate their *J*_*1*_ by considering the future chance of reviewing their judgment (*J*_*2*_) with the knowledge of *J*_*S*_, we instructed them that the chance to review would be given only in some trials. The participants did not have a chance to revise for 8 randomly arranged trials among 60 trials. We analyzed 52 valid trials per participant. Critically, we instructed the participants that they did not need to change their *J*_*1*_, but that they could if they felt that the changes would be more preferable judgments.

A fixation cross was shown for the inter-stimuli intervals (ISI) after the scenario presentation, and between judgments (*J*_*1*_, *C*, and *J*_*2*_), and for the inter-trial interval (ITI). Both ISI and ITI durations were randomized from 4 s to 7 s. We presented scenarios in pseudo-random order across participants. Specifically, the order of presentation of conditions of the different size jury group and its combination with sets of scenarios were counterbalanced across subjects.

### Judgment of other jurors and social contextualization

We instructed the participants that the given social information, J_S_, is the average judgments of some of the previous participants. Specifically, we informed them that the computer randomly selected the judgments of 4 or 19 previous participants according to the condition.

Moreover, participants were told that only those individuals who had assigned a higher level of confidence to their initial judgments (*J*_1_) than the average level of confidence of all participants were selected. Likewise, participants were informed that their judgments would be presented to the next participants when they reported a high level of confidence. Using this design, participants were explicitly informed that the other jury members would be different in each trial.

J_S_ was a computer-generated value. We manipulated the judgments of others to make sure that all participants reconsidered their judgments under influences of all ranges of differences in judgments (|*D*|). Notably, by design, the group never had the same level of punishment judgments as individual participants (*J*_1_ ≠ *J*_*S*_). Simultaneously, we ensured that the judgments of the group were not too different from those of participants’ in order to make participants believe that those were made by other previous participants. To do this, J_S_ was established for each trial by the computer so that the difference (*D* = *J*_*S*_ − *J*_1_) between J_S_ and J_1_ was within the range of 4 years to 10 years (4 ≤ |*D*| ≤ 10).

Moreover, to control for the possibility that participants might be able to learn the consistency of the group judgments (social information) over the trials, the direction of differences in judgments (whether social information was severer or milder than one’s initial judgments, J_1_) was pseudo-randomly determined across trials. That is, we ensured that every participant was given judgments of others that were more severe than theirs during one half of trials and that judgments of others were more lenient than theirs during the other half.

On average, the participants perceived 6.98 ± 0.18 (SEM) years difference from their initial judgments when confronted with the judgments of others. This was also true within participants while they were making judgments within different jury group sizes (*G*) ($\left|\bar{D}\right|=\;7.10\;\pm \;0.25$ (when *n* = 20; $\left|\bar{D}\right|=\;6.85\;\pm \;0.27\;$ when *n* = 5).

### The scenarios

A crime scenario was composed of the facts (plain explanation of who did what) and the circumstances (contexts and reasons why the defendant committed the murder). One hundred murder-case scenarios were initially produced for this study, which included 32 cases collected from a previous study [67] and 68 additional scenarios inspired by real stories taken from the news with the same structure.

The length ranged from 50 to 60 words (mean length = 54.95 ± 0.36 [SEM]). All scenarios were written in 3 sentences. To minimize the bias, we informed participants before the experiment that we changed the name of the defendants to either “Jean” or “Marie.”

Half of the scenarios included the circumstances that were expected to induce sympathetic emotion for the defendant (sympathetic cases), and the other half did not (nonsympathetic cases). Sixty among 100 scenarios were selected based on the responses of elicited sympathetic emotions that were acquired from the 20 different healthy subjects (10 males, mean age = 21.43 ± 0.46 [SEM] years) using the same scale of sympathetic emotion ratings. The average rating for selected scenarios was −0.48 ± 0.21 (SEM). They were significantly split into 2 groups according to their rating (*t* = −39.31, *P* < 0.001, 2-sample *t* test). The duration of scenario presentation in the main experiments (15 s) was also decided based on the sample group’s speed of reading.

### Behavioral data analysis

The impact of social information, modulating individual judgments, was measured at the level of conformity (LC).

$$LC=\frac{J_{2}\;-\;J_{1}}{J_{S}\;-\;J_{1}}$$ (1)

We also compared them under the impacts of different levels of subjective confidence (*C*, individual median split) and different group sizes (*G*). All the comparisons were performed by LMEM.

### Bayesian decision-making model

The Bayesian decision-making model assumed that participants were trying to estimate the most preferable punishment judgment (*J*) for each scenario. There were 2 cues that participants could rely on: (1) their initial reading of the scenario, which led to a private estimation of individuals (*j*_*Individual*_) of *J*, and (2) the estimation made by the social group (*j*_*Social*_) of *J*. Participants combined these 2 cues to produce a final estimate of *J* where the value of *J* maximized the probability distribution, *p*(*J*|*j*_*Individual*_, *j*_*Social*_). Applying Bayes rule, we evaluated the probability distribution as

$$p\left(J|j_{Individual},\;j_{Social}\right)=p\left(j_{Individual},\;j_{Social}|J\right)\times p\left(J\right)/p\left(j_{Individual},\;j_{Social}\right)$$

Note that in the above equation, *p*(*J*) was the prior belief about *J* (i.e., the belief about *J* even before receiving any information). In the current study, this prior belief was considered to have uniform distribution (no biases). By considering the social and the individual estimates to be independent in this study, we could estimate the *p*(*j*_*Individual*_|*J*) as below.

$$p\left(J|j_{Individual},\;j_{Social}\right)=p\left(j_{Individual}|J\right)\times p\left(j_{Social}|J\right)\times p\left(J\right)/p\left(j_{individual},\;j_{Social}\right)$$

The *p*(*j*_*Individual*_|*J*) was assumed to be Gaussian, ~ *Norm*(*J*_1_, *τ*^2^), with mean, *J*_*1*_, and variance, *τ*^*2*^. The credibility of the individual estimate was thus 1/*τ*^2^, which was dependent on the level of confidence reports.

$$p\left(j_{Individual}|J\right)\;\sim \;Norm\left(J_{1},\;\tau^{2}\right)\;where\;\tau^{2}={\left(\beta_{C}+\omega_{C}C\right)}^{-1}$$ (2)

Similarly, we considered *p*(*j*_*Social*_|*J*) to be Gaussian, ~ *Norm*(*J*_*S*_, *σ*^2^), with mean, *J*_*S*_, and variance, *σ*^*2*^.

$$p\left(j_{Social}|J\right)\;\sim \;Norm\left(J_{S},\;\sigma^{2}\right)$$ (3)

The credibility of the social information *p*(*j*_*Social*_|*J*) was thus 1/*σ*^2^, which was dependent on the group size. Specifically, when a participant was confronted with the judgments of groups of different sizes, she might assign a different level of credibility to social information (e.g., the credibility $1/\sigma_{20}^{2}$ was assigned to *j*_*Social*_ when the group was large; the credibility $1/\sigma_{5}^{2}$ was assigned to *j*_*Social*_ when the group was small).

Inter-individual differences in estimated credibility of social information were measured by their ratio, called “relative credibility” (*Δσ*). This parameter *Δσ* indicated, therefore, the individual variability of the sensitivity to the changes in group size when participants assigned credibility to social information when confronted with the judgments of others (*J*_*S*_).

$$\Delta \sigma =log\left(\frac{1/\sigma_{20}^{2}}{1/\sigma_{5}^{2}}\right)=log\sigma_{5}^{2}-\;log\sigma_{20}^{2}$$ (4)

When the prior belief, *p*(*J*), was given as a uniform distribution, the final distribution, *p*(*J*|*j*_*Individual*_, *j*_*Social*_), also followed a normal distribution with its mean being the weighted average of these 2 cues. Each cue was weighted by its credibility, and the credibility of the combined belief was the sum of these 2 levels of credibility.

$$p\left(J|j_{Individual},\;j_{Social}\right)\;\sim \;Norm\left(\frac{\tau^{2}J_{S}+\;\sigma^{2}J_{1}}{\sigma^{2}+\tau^{2}},\;\frac{\sigma^{2}\tau^{2}}{\sigma^{2}+\tau^{2}}\right)$$ (5)

Eventually, participants who made the initial judgments, *J*_*1*_, based on their individual estimates of *J* (*p*(*j*_*Individual*_|*J*)) changed to *J*_*2*_ based on their final estimates of *J* (*p*(*J*|*j*_*Individual*_, *j*_*Social*_)). According to that, the Bayesian model predicted the judgments, $\hat{J_{2}}$. Specifically, changes in judgments, $\hat{J_{2}}-J_{1}$, were predicted by the Kullback–Leibler divergence (*D*_*KL*_) between 2 probability distributions—the individual estimate of *J*, *p*(*j*_*Private*_|*J*), and the final estimate of *J*, *p*(*J*|*j*_*Individual*_, *j*_*Social*_). The value of *D*_*KL*_ was computed as [40,68]

$$\hat{J_{2}}-J_{1}=D_{KL}\left(p\left(j_{Individual}|J\right)||p\left(J|j_{Individual},\;j_{Social}\right)\right)=\sum_{i=0}^{30}p\left(i|j_{Individual}\right)\times \text{log}\frac{p\left(i|j_{Individual}\right)}{p\left(i|j_{Individual},\;j_{Social}\right)}$$ (6)

where *p*(*i*|*j*_*Individual*_) was the probability that the punishment magnitude of *i* years would be made when the juror had an individual estimate of *J*, *p*(*j*_*Individual*_|*J*) as the preferable punishment for the defendant in the scenario, and *p*(*i*|*j*_*Individual*_, *j*_*Social*_) was the same quantity, given the final estimate of *J*, *p*(*J*|*j*_*Individual*_, *j*_*Social*_) after integrating social information *p*(*j*_*Social*_|*J*).

### Alternative models of judgment adaptation

We also predicted changes in judgments ($\hat{J_{2}}$) under social influences with 2 alternative models. Together with the prediction of the “Bayesian model” (Eq 6), these predictions ($\hat{J_{2}}-J_{1}$) of alternative decision-making models—the “linear model” (Eq 7) and “surprise model” (Eq 8)—were compared with actual behavioral changes (*J*_2_ − *J*_1_).

$$\hat{J_{2}}-J_{1}=\beta_{L}+\left(\omega_{D}+\omega_{G}G+\omega_{S}S\right)D+\omega_{C}C+\omega_{DC}DC+\omega_{J}\left|J_{1}\right|$$ (7)

The “linear model” (Eq 7) predicts the changes in judgments ($\hat{J_{2}}-J_{1}$) by a linear regression model that takes into account the given deviation in the group judgments (*D = J*_*S*_ *− J*_*1*_), the level of confidence that one had for their judgments (*C* = [−1:+1]), the group size (*G* = [0, small group trials; 1, large group trials]), and 2-way interaction effects (*D* × *C*, and *D* × *G*). We also assumed an interaction effect between the deviation in judgments and the types of scenarios (*D* × *S* where *S* = [−1, sympathetic cases; 1, nonsympathetic cases]). That is, participants were expected to change their judgments more when the group made milder level of punishment than themselves (*J*_*S*_ < *J*_*1*_) in sympathetic scenarios and when groups made severer punishment than themselves (*J*_*S*_ > *J*_*1*_) in nonsympathetic scenarios. We also tested the effect of initial judgments (*J*_*1*_) to test its potential effect on regression-to-the-mean: the behavioral tendency that participants were more (or less) likely to conform to *J*_*S*_ when *J*_*1*_ was close to extreme (0 years or 30 years). To test this effect, we included the absolute value of the normalized *J*_*1*_ (−1 to 1 range; |*J*_*1*_ |) in the regression analysis. Therefore, |*J*_*1*_ | indicated the scale of judgments that participants moved from the default (15 years) while making the initial judgment. In this model, *β*_L_ indicates the constant. Taken together, the “linear model” tested whether individuals were motivated to reduce the perceived deviation in the group judgments by adjusting their prior judgments. In particular, this model also tested whether this behavioral tendency was stronger when they had high confidence in their judgments, when they were confronted by judgments of the large group, and when they were confronted by judgments of the group that were more extreme than theirs.

$$\hat{J_{2}}-J_{1}=\beta_{S}+\omega_{U}U+\left(\omega_{G}G+\omega_{S}S\right)D+\omega_{J}\left|J_{1}\right|$$ (8)

The “surprise model” (Eq 8) predicts the changes in judgments ($\hat{J_{2}}-J_{1}$) by a linear regression model that also takes into account the interaction effects with group size and scenario types (*D* × *G*, and *D* × *S*), and the initial judgments (*|J*_*1*_ *|*), which were defined the same as those in the “linear model.” One difference of the “surprise model” is that it considers confidence as a level of precision of one’s own belief about the right amount of punishment for the criminal. Therefore, this model assumes that participants change their judgments based on the surprise of how much the judgment of others differs from their own belief. According to information theory [40], the surprise (*U*) evoked by such unpredicted observation is the entropy of the unpredictability of an event, *α*, given the belief of the participant

$$U=-logp(\alpha |Prior))=-log\;p(J_{s}|Norm\left(J_{1},\;\tau^{2}\right))$$ (9)

where τ follows a function of one’s reported confidence as in Eq 2 in the “Bayesian model,” which includes 2 free parameters (*β*_C_ and *ω*_C_). This model predicts that changes in judgments are proportional to how unlikely others made their judgment, *J*_*S*_, given the prior beliefs of participants, *J*_*1*_, with the subjective confidence, *C*.

### Behavioral model comparison

The model predictions of the changes in judgments ($\hat{J_{2}}-J_{1}$) were fitted with subjects’ changes in behavioral performance(*J*_2_ − *J*_1_). We estimated the parameters that maximized the predictability of each of the 3 models. The goodness-of-fit of each model was measured with its log likelihood. If the t × 1 vector of observation is denoted by X (t indicates the number of trials) and its prediction is estimated as the vector $\hat{\text{X}}$ (same length as X), the relationship between the sample and its prediction is defined as:

$$X=\eta \hat{X}+\varepsilon$$

The vector of the error term, ε, has a multivariate normal distribution conditional on η. We assumed that the mean of the error distribution was equal to 0 and that the covariance was equal to ϵ^2^.

$$\epsilon^{2}=Var[\varepsilon_{i}|\eta ]$$

The log likelihood of each model is equal to the logarithm of the product of the likelihoods of each change in judgment. Therefore, the −2log likelihood function is computed as follows:

$$-2lnL\left(X,\hat{X}\right)=t\;ln\left(2\pi \epsilon^{2}\right)+\frac{1}{\epsilon^{2}}\sum_{i=1}^{t}{\left({\hat{X}}_{i}-\eta X_{i}\right)}^{2}$$ (10)

To account for over-fitting, we trained each of the models using an in-sample optimization procedure using an iterative LOOCV procedure, in which data from all but 1 trial (51 trials) were used to perform an out-sample prediction against the left-out trial data of each subject. This procedure was repeated 52 times by omitting a different trial each time. Therefore, data from each of the 52 trials was used exactly once as validation data. The series of log likelihoods were averaged across trials and across participants to produce a single estimation. By comparing the prediction power of our models, we examined which model could best explain the underlying mechanisms of the process of judgment adaptation. Model parameters were fitted using the multivariate constrained minimization function in MATLAB 2015 (MathWorks, MA, USA). To select the best model among the 3, we compared the log likelihood that introduced a penalty according to the number of free parameters. The BIC is computed based on the log likelihood where k is the number of estimated parameters in the model.

BIC=−2lnL+kln(t) (11)

We assumed that the model parameters were fixed throughout execution of the task, because subjects had been instructed that each murder case was independent and also because participants were told that the other members of the jury differed on each trial. The number of free parameters of each model was as follows: (1) 7 for the “linear model”—ω_D_, ω_G_, ω_S_, ω_C_, ω_DC_, ω_J_, and β_L_; (2) 7 for the “surprise model”—ω_U_, ω_G_, ω_S_, ω_J_, ω_C_, β_C_, and β_S_,; and (3) 4 for the “Bayesian model”—β_C_, ω_C_, σ^n = 5^, and σ^n = 20^.

### Neural model comparison

We also compared the neural correlates of the model predicting changes in judgment with the corresponding brain responses encoding changes in judgments. We ran a separate GLM using parametric regressors of changes in judgement and the model predictions of these changes (GLM3). We extracted the parameter estimates representing the neural correlates of each model prediction. We then compared them with brain responses that correlate with behavioral changes in judgment. Parameter estimates were extracted from the dACC ROI. Consistent with neural model comparisons from previous studies, we compared the likelihood of each model [69,70]. When the variance of errors of each model prediction is ϵ^2^, and t is the number of time series, the −2 log likelihood is computed as described by Eq 10. All models had 1 parameter, the coefficient of the single regressor.

### fMRI data acquisition

Functional images were acquired with a 1.5 T Siemens Magnetom Sonata Maestro Class MRI System (Siemens, Munich, Germany) at the CERMEP in the Groupement Hospitalier Est, Lyon, France. A higher order shimming procedure was completed covering the whole brain of each participant. The imaging parameters for the EPI T2*-weighted sequence were as follows: repetition time (TR), 2500 ms; echo time (TE), 60 ms; flip angle, 90°; FOV, 220 mm, acquisition matrix, 64 × 64, slice thickness = 4 mm. Contiguous slices were acquired in interleaved order. To acquire whole-brain images, the magnetic field was tilted with minus 20° from the anterior to posterior commissure line (AC-PC) of each participant. The imaging parameters for the T1-weighted anatomical scan were as follows: TR, 1970 ms; TE, 3.93 ms; FOV, 256 mm; matrix 256 x 256; slice thickness, 1 mm.

The stimuli were presented with a screen resolution of 1024 × 768 pixels, displayed at a visual angle of 24 × 18°, centered on a 500 × 500 pixel array, and surrounded by a black background. The participants were asked to use their index and middle fingers of both hands to answer by pressing a 4-button controller. Stimuli were presented, and the responses to the stimuli were collected using the software Presentation (Neurobehavioral Systems, CA, USA).

### fMRI data analysis

Image preprocessing was performed using SPM8 (Wellcome Trust Centre for Neuroimaging, UCL, UK). Time-series images were registered in a 3-dimensional space to minimize any effect that could result from the motion of the participants’ heads. Functional scans were realigned to the last volume, corrected for slice timing, and unwarped to correct for geometric distortions. Inhomogeneities, distortions related to correction maps, were created using the phase of nonEPI gradient echo images measured at 2 echo times (5.19 ms for the first echo and 9.95 ms for the second). These were coregistered with structural maps, spatially normalized into the standard Montreal Neurological Institute (MNI) atlas space, and then spatially smoothed with an 8 mm isotropic full-width at half-maximum (FWHM) Gaussian kernel using standard procedures in SPM8.

We constructed 3 separate GLMs. For the first GLM (GLM1), we ran a first-level analysis, modeling brain responses related to revising judgments while confronted with the judgments of others. The conformity trials (LC > 0) and nonconformity trials (LC ≤ 0) were modeled separately. They were modeled as a boxcar function time-locked to the onset of social information (*J*_*S*_) with the duration of a response time in each trial to make a judgment (*J*_*2*_). Brain responses related to making punishment judgments after reading a crime scenario (*J*_*1*_) were modeled separately. These were modeled as a boxcar function time-locked to the onset of decision-making with duration of reaction times in each trial. In addition, the 6 motion parameters produced for head movement and the 2 motor parameters produced for buttons pressing with the right and the left hands were also entered as additional regressors of no interest to account for motion-related artifacts. All these regressors were convolved with the canonical hemodynamic response function. Contrast images were calculated and entered into a second-level group analysis. In the GLM1, brain regions recruited by conformity decisions were first identified using the contrasts “conformity trials > nonconformity trials.”

We also tested another version of GLM1 with a fixed boxcar duration of 4 s starting at the onset of social information (*J*_*S*_) with all other settings kept identical to GLM1. By fixing the boxcar duration, we tested whether the estimated brain activity in GLM1 was still robust when disregarding inter-trial differences in responses times that could reflect different levels of difficulty in decision-making. To do this, we compared the effects sizes of GLM1 to those of an alternative model. If the effect size estimated from the GLM1 does not decrease in this alternative model, this supports the idea that brain activity observed in GLM1 is involved in judgment adaptation rather than processing the level of difficulty.

The second GLM (GLM2) was the same as GLM1 except that the blood-oxygen-level dependent (BOLD) response related to revising judgments was separately modeled by group size, instead of conformity decisions. In detail, BOLD responses related to revising judgments when confronted by the judgments of a larger group (20 juries) were separately modeled from those related to revising judgments when confronted with the judgments of a smaller group (5 juries). They were further modulated by parametric regressors accounting for a perceived difference in judgments (*D* = *J*_*S*_ − *J*_*1*_). Contrast images were calculated based on the parameter estimates output by the GLM and were then entered into a second-level group analysis. First, we found the group-size–related brain activity (large group trials > small group trials). Second, we found the brain area where the group-size–related activity further correlated with individual differences of *Δσ*, which was defined as the ratio between the estimated credibility of social information in the large group and the credibility of social information in the small group. To this end, we performed a regression analysis in the second-level analysis by entering *Δσ* as a covariate across participants.

The third GLM (GLM3) was designed to identify the brain regions in which activity parametrically encoded the model-based predictions of changes in judgments that were estimated by the 3 different computational models ($\hat{J_{2}}-J_{1}$) and the actual behavioral of changes in judgments (*J*_2_ − *J*_1_). For this GLM, each event was treated as a regressor to extract the time series of beta parameters. This approach has previously been used for multivariate and functional connectivity analyses [71]. In GLM3, the BOLD responses related to revising judgments were modeled as a boxcar function including reaction times and modulated by parametric regressors accounting for the 3 different model predictions. Again, because differences in reaction times may reflect inter-trial differences in the level of difficulty in decision-making, we also tested an alternative model of GLM3 by fixing the boxcar duration to 4 s starting at the onset of social information (*J*_*S*_). All the other settings were kept identical with the GLM3.

We compared the parameter estimates related with the model-based predictions of changes in judgments to find which of them closely represents the parameter related to actual changes in judgments. BOLD responses to the initial judgments (*J*_*1*_) and 8 types of motion regressors were also included in the GLMs and convolved with the canonical hemodynamic response function (HRF). Modulation of brain activity by the changes in judgments and the model-based predictions of changes in judgments were calculated and entered into a second-level analysis. We further examined how accurately the behavioral models explained BOLD responses related to changes in judgments. For this analysis, we extracted the time courses of dACC activity from the first-level contrasts of each participant modulated by model prediction of changes in judgments. They were then compared with the time course of dACC activity modulated by the actual changes in judgments. All time courses were extracted from the dACC ROI that we defined as a 10-mm diameter sphere, centered on the dACC ROI at x,y,z = 8,18,46, based on a previous meta-analysis study [28]. The accuracy level of BOLD activity was estimated by the −2 log likelihood of all participants (Eq 10) and the BIC, considering the number of free parameters of each model were used for the model comparison.

To investigate whether dACC activity reflects Bayesian updates rather than choice difficulty, we performed an additional GLM (GLM4). With this model, we addressed the alternative interpretation that the variance in decision-related dACC activity reflects how difficult the decision was and how long it took. As in GLM3, the BOLD responses related to revising judgments were modeled with a 4-s boxcar duration, starting at the onset of social information (*J*_*S*_). We included the following parametric regressors: the Bayesian updates measures (*D*_*KL*_ in Eq 6), the level of unexpected surprise (*U* in Eq 9), as a measure of difficulty, and the reaction times. In doing so, we examined whether the Bayesian update regressors could still explain dACC activity when allowed to compete with other regressors that possibly explain variance in the dACC signals. In the model specification process, the serial orthogonalization of parametric modulators was turned off. A SVC was performed within the dACC cluster activated for the conformity decision (*P*_FWE_ < 0.05; on the basis of an initial uncorrected threshold at *P* < 0.001).

We report results corrected for FWE with multiple comparisons (*P*_FWE_ < 0.05). This approach assesses the strength of activations defined by an initial uncorrected threshold, which we take as *P* < 0.001 for all analyses [72].

### ROI analysis

Three ROIs were defined independently to test and to support our findings. They include dACC and right and left lateral FPC. For each analysis, a single, predefined ROI was used. For the ROI analyses, a SVC was performed (P_FWE_ < 0.05; on the basis of an initial uncorrected threshold at *P* < 0.001 [72]).

We used an a priori anatomically defined region of the dACC, which was defined by a 10-mm diameter spherical ROI centered at x,y,z = 8,18,46 (MNI coordinates). This dACC ROI was adopted based on the results of an ALE meta-analysis concerning the neural substrates of conformity behavior [28]. To correct the results of the conformity-decision–related activation for multiple comparisons (GLM1), we used a SVC. The same ROI was also used to extract beta parameter estimates to compare computational models to the dACC activity representing the changes in judgments (GLM3). Parameter estimates were extracted from all voxels in the ROI and averaged using MarsBaR 0.43 (http://marsbar.sourceforge.net). Finally, this ROI was used to test whether its functional connectivity to the right lateral FPC was modulated by the changes in group sizes.

We defined 2 ROIs in the bilateral FPC to extract parameter estimates. The beta parameters were extracted from 2 FPC ROIs, defined as 10-mm diameter spheres centered at each peak voxel of the clusters in the bilateral FPC (x,y,z) = (27,57,10) and (−30,58,15) in MNI coordinates.

To further check that the recorded pattern of FPC activity reflected individual differences, we used 2 additional analyses [73,74]. First, the changes in the parameter estimate (large group trials—small group trials) served to perform a bootstrapping sampling analysis (10,000 times of iteration). Notably, parameter estimates and bootstrapping were performed separately from the ROIs in the right and the left FPC. This procedure allowed us to infer the effect size of the relationship between the changes in FPC activation and sensitivity to group size when assigning credibility to social information (Δσ) across individuals in a larger dataset. The relationship between the vector of the predicted parameter and the same length vector of the extracted parameter estimates were tested with a linear regression, which enabled us to estimate mean predictability and standard deviation. Based on an assumption that the predictability distribution is normal, we further estimated the 95% confidence interval. Second, we performed a scrambling analysis [75,76]. That is, we scrambled Δσ across participants and assigned each participant’s Δσ to another participant. In doing so, we tested whether any brain activity represents this randomly assigned Δσ.

### Psychophysiological interaction analysis

To assess changes in functional connectivity during the presence of social information as a function of changes in group size (large, 20-person jury versus small, 5-person jury conditions), we performed a PPI analysis. The PPI allowed us to identify the brain areas where activity can be explained by the interaction between activity in a seed region and the subsequent process involved in the decisions to conform to social information. For the PPI, we defined the seed region in right FPC on the peak voxel, (x,y,z) = (41,44,19). We used the Generalized PPI toolbox from SPM (gPPI) [77], which allowed us to create a new GLM in which the deconvolved activity of the seed region is assigned to the context-dependent regressors and reconvolved with HRF. Average time courses were extracted from all voxels in a predefined ROI surrounding the peak voxel in the right FPC clusters. The time courses were extracted from the activities modulated by group size (GLM2 at the contrast of “large-group trials > small-group trials”). The main effects of trials in different group sizes, the seed-region time course, and motion parameters were included as regressors of no interest. The PPI contrast compares large-group trials × right FPC (+1) with small-group trials × right FPC (−1).

## Supporting information

S1 Fig Dorsal anterior cingulate cortex (dACC) activity when changing judgments to conform to those of the group. The peak is located at (x,y,z) = (0,12,39). The HRF was convolved with a boxcar of 4 s after the onset of social information (alternative GLM1; P_FWE_<0.05 in SVC).
(PNG)

S2 Fig Activation map showing the parametric effects of the changes in judgments predicted by the Bayesian model (the KL divergence). The dACC region was found to compute the level of judgment updates that was predicted by the Bayesian model, even in a situation where the level of surprise and reaction times were included as additional regressors. The peak is located at (x,y,z) = (6,8,49; GLM4; P_FWE_<0.05 in SVC).
(PNG)

S1 Data Data for Figs 1C, 1D, 3A, 3B, 3C, 3D, 4B, 5B and 6. (XLSX)

## Abbreviations

BIC: Bayesian information criteria
dACC: dorsal anterior cingulate cortex
fMRI: functional MRI
FPC: frontopolar cortex
FWE: family-wise error
GLM: general linear model
IPL: inferior parietal lobule
LMEM: linear mixed-effects modeling analysis
LOOCV: leave-one-out-cross-validation
PPI: psychophysiological interaction
ROI: region of interest
SEM: standard error of the mean
SVC: small volume correction
vmPFC: ventromedial prefrontal cortex
