# Supplementary figures and images for: Integration of individual and social information for decision-making in groups of different sizes
Source: PLoS Biol. 2017 Jun 28;15(6):e2001958. doi: 10.1371/journal.pbio.2001958 (PMC5489145; doi:10.1371/journal.pbio.2001958)

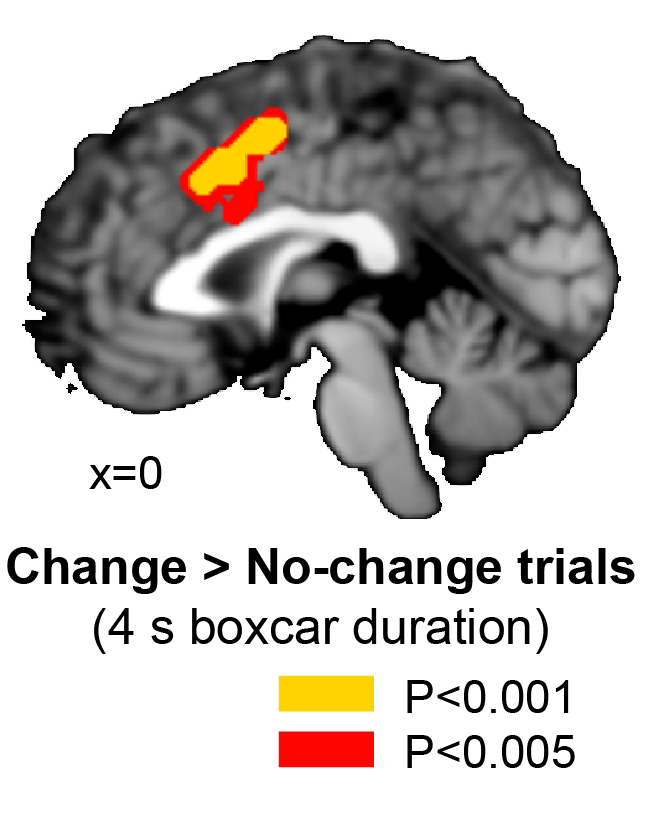

Supplement: S1 Fig — The peak is located at (x,y,z) = (0,12,39). The HRF was convolved with a boxcar of 4 s after the onset of social information (alternative GLM1; PFWE<0.05 in SVC). (PNG) [file pbio.2001958.s001.png]

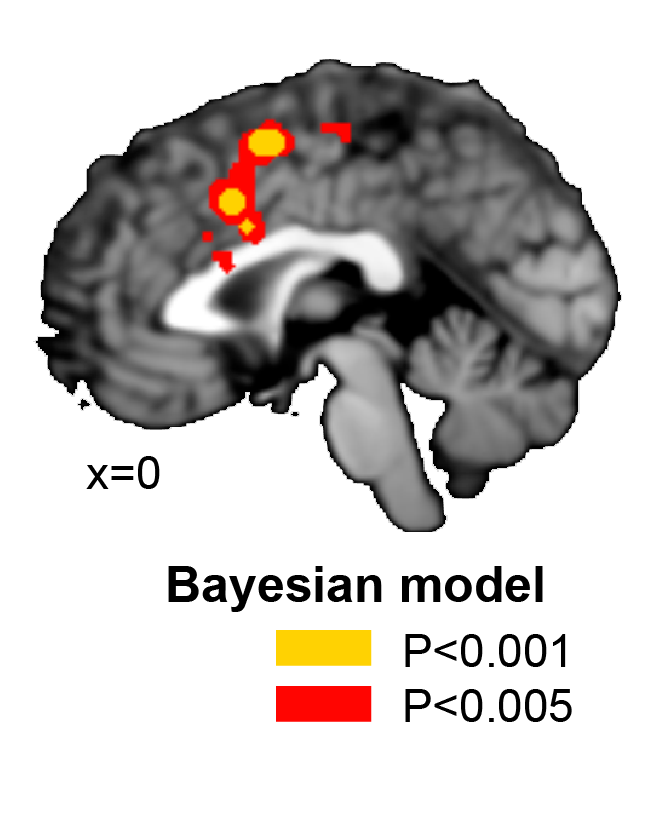

Supplement: S2 Fig — The dACC region was found to compute the level of judgment updates that was predicted by the Bayesian model, even in a situation where the level of surprise and reaction times were included as additional regressors. The peak is located at (x,y,z) = (6,8,49; GLM4; PFWE<0.05 in SVC). (PNG) [file pbio.2001958.s002.png]
